# Subcutaneous adipose tissue alteration in aging process associated with thyroid hormone signaling

**DOI:** 10.1186/s12920-023-01641-5

**Published:** 2023-08-25

**Authors:** Wen-Na Zhang, Hao Zhu, Zhen-Wu Ma, Jing Yu, Yun Yang, Xuan-Bei Lu, Yi-Fan Lv, Xiao-Dong Wang

**Affiliations:** 1 Division of Endocrinology, the First Affiliated Hospital of Nanjing Medical University, 300 Guangzhou Road, Nanjing, 210029 China; 2 Division of Geriatric Endocrinology, the First Affiliated Hospital of Nanjing Medical University, 300 Guangzhou Road, Nanjing, 210029 China

**Keywords:** Chromatin accessibility, Subcutaneous adipose tissue (SAT), Thyroid hormone, Aging, Mitochondria

## Abstract

**Background:**

Functional changes in subcutaneous adipose tissue (SAT) occur earlier in the aging process and play an important role in the occurrence and development of age-related metabolic diseases. The mechanism of this phenomenon is still unclear, and the change in adipose tissue with age is poorly understood.

**Methods:**

We used transcriptome sequencing (RNA seq) to screen differentially expressed genes at the mRNA level, and analyzed the functional characteristics of the differential genes through GO and KEGG analysis in human SAT of all ages. In order to clarify the specific mechanism of the functional change, we analyzed the chromatin accessibility in the promoter region in the same SAT used in the RNA seq by the assay for transposase-accessible chromatin with high throughput sequencing (ATAC-seq) and obtained the functional genes in SAT changed with age. To verify these changes, we enlarged our sample content of human SAT. The primary mice adipocytes were extracted and stimulated by thyroid hormone of different concentration to construct an animal model, and the expression of the genes were determined through real-time Polymerase Chain Reaction(RT-PCR). The oxygen consumption test and immunofluorescence staining were used to determine the mitochondrial function of SAT.

**Results:**

RNA-seq showed characteristic gene expression of young and old human SAT, in which 331 genes were up-regulated and 349 genes were down-regulated. ATAC-seq, RNA-seq, combined with the mouse prediction model, determined the functional changed characteristics of seven genes. All these genes expressed differently in SAT of different ages, in which, NCF1, NLRP3, DUOX1 showed positive correlation with age; The expression of IFI30, P2RX1, P2RX6, PRODH, however, decreased with age. And all these genes showed dose dependent alternations under treatment of triiodothyroxine in mice SAT. The oxygen consumption rate revealed significant changes of the mitochondrial function and ROS accumulation in human SAT of different ages.

**Conclusion:**

In elderly individuals, the function, in addition to distribution, of SAT undergoes significant changes, primarily in mitochondria, which may be due to insensitivity to thyroid hormone signaling. These results identified seven novel genes regulated by thyroid hormone, exhibiting significant changes in SAT of different age, and are probably related to the dysfunction of the aged SAT due to the mitochondrial damage and ROS accumulation.

**Supplementary Information:**

The online version contains supplementary material available at 10.1186/s12920-023-01641-5.

## Impact statement

Aging is characterized by many profiles, changes of adipose tissue (AT) included. In this article we seek to clarify the change of subcutaneous adipose tissue (SAT) during aging and the possible cause behind it, which might provide us with new intervention methods against aging. Many studies have been done on AT, yet few focused on the relationship between thyroid and SAT, combining the newly applied ATAC-seq and RNA-seq, we demonstrated the mitochondrial and ROS associated functional changes of SAT in the process of aging. And within the SAT of the young and the elderly, we identified seven genes whose expression are dramatically altered and all of their chromatin open regions contain thyroid hormone binding site, further studies were carried out and proved that they do can be stimulated by thyroid hormone. Thus, providing us new sights into SAT functional changes during aging, and possible intervention targets against senescence.

## Background

The world population is aging along with the increasing proportion of obesity. By 2030, people over 65 years old will comprise 20% of the US population. By 2035, 25% of Europe’s populace will be 65 or older [1]. In the past 40 years, the proportion of obesity has tripled in the elderly population [2]. Many studies have indicated a positive relationship of body mass index (BMI) with chronic diseases and mortality in young and middle-aged individuals. However, evidence shows that in elderly individuals, the correlation between mortality and BMI is relatively negative, and the optimal BMI of elderly individuals ranges from 21.0 to 31.9 kg/m^2^, which is known as the “obesity paradox” [3]. The mechanism of this phenomenon is still unclear because the change in adipose tissue with age is currently poorly understood.

In advanced old age, although the total amount of adipose tissue tends to increase, its distribution dramatically changes [4]. To elucidate whether these differences are due to anatomic location or intrinsic differences in adipose depots, several studies have investigated mice after transplantation of subcutaneous or visceral obesity from donor mice into either subcutaneous or visceral regions of recipient mice; Through transplantation of the subcutaneous fat into visceral compartments, the recipient mice demonstrated decreased body weight and total fat mass and improved glucose metabolism and insulin sensitivity during hyperinsulinemia, whereas other study show that the transplantation of visceral fat performed an adverse effect on glucose homeostasis [5]. The major resulting effects on metabolism and body weight are beneficial effects due to the added subcutaneous fat rather than a detrimental effect of visceral fat [6, 7], and that this effect is at its greatest when the subcutaneous fat is placed in an intra-abdominal site [5, 8]. Therefore, adipose tissue itself is particularly important for metabolic diseases. By conducting age-related transcriptional profile sequencing of 17 mouse organs, a previous study has reported that genes related to SAT and gonadal adipose tissue are activated in the transcriptional profile of significant aging-related differential genes before genes related to other organs when the mice are 12 months old (similar to 36 years old in humans), indicating that SAT appears earlier in the aging process [9]. Thus, these findings suggest that SAT alteration in the aging process may be related to aging-related metabolic diseases. However, it is unclear which aging-related changes occur in SAT.

Thyroid hormone (TH) regulates metabolic processes essential for normal growth and development as well as metabolism in adults. With increasing age, the metabolic capacity of elderly individuals decreases, but the thyroid hormone level does not change significantly, which may be due to the decrease in the sensitivity of elderly individual to thyroid hormone [10]. It has been reported that different target organs show different sensitivities to thyroid hormone [11]. Adipose tissue, as one of the most important target organs of thyroid hormone, is capable of modulating physiological functions by synthesizing and releasing adipokines, such as adiponectin [12–14]. T3 and adiponectin play important roles in controlling normal metabolic functions. Therefore, we hypothesized that the generation of aging-related diseases may be related to the regulation of adipose tissue function by thyroid hormone.

In the present study, we conducted RNA-seq analysis on human SAT of different age groups with normal thyroid function. The results of gene sequencing suggested that aging SAT undergoes significant changes, including DNA damage, inflammation, fibrosis, mitochondrial damage, mitochondrial dysfunction, and ROS accumulation. Measurement of O_2_ consumption and immunofluorescence staining performed on human tissues confirmed that aging SAT shows mitochondrial damage and ROS accumulation. To explore the aging mechanism of SAT, we also performed ATAC-seq detection [15–17] on the same samples used in RNA-seq, which demonstrated that many gene open regions in aging subcutaneous adipocyte genes have thyroid hormone-related receptors and their co-transcription factor-binding sites. In both mouse and human models, we identified seven genes related to mitochondrial function and ROS accumulation change with age, and these seven genes are regulated by thyroid hormone. Therefore, we speculated that the aging-related changes in SAT may be linked with the altered effect of thyroid hormone signaling on target genes, demonstrated as mitochondrial dysfunction and enhanced oxidative stress response, leading to related metabolic diseases.

## Methods

### Participants

In patients undergoing cholecystectomy, SAT was collected from 3 young men (aged 32.67 ± 0.94) and 3 old men (aged 67.33 ± 1.70) with normal thyroid function including normal Free thyroxine (FT4 12.0–22.0 pmol/L), normal Free triiodothyronine (FT3 3.10–6.80 pmol/L), normal thyroid-stimulating hormone(TSH 0.27–4.2 mIU/L) and body mass index (BMI ). When selecting patient samples, patients with malignant tumors and other metabolic diseases were excluded, and patients with a history of taking drugs affecting lipid metabolism were also excluded. The SAT was collected from the navel side of the midabdomen, immediately placed into liquid nitrogen, and transferred to -80 °C for storage. The characteristics of the study group are shown in Supplemental Tables 1, and whole genome analysis was performed on all samples.

### Total RNA extraction

We used TRIzol (Invitrogen, Carlsbad, CA, USA) to extract the total RNA following to the manufacturer’s instructions. Tissue weigh approximately 60 mg in liquid nitrogen was pestled into powder in a 2 mL tube, then homogenized for 2 min, followed by 5 min rested horizontally. The mixture was centrifuged at 12,000×g at 4 °C for 5 min, and the supernatant was then transferred into another EP tube containing 0.3 mL of chloroform/isoamyl alcohol (24:1). The mixture was shaken vigorously for 15 s and centrifuged for 10 min at 12,000×g at 4 °C. After centrifugation, the upper aqueous phase containing the RNA was transferred into another tube with an equal volume of supernatant of isopropyl alcohol, after which, the mixture was put under centrifugation at 13,600 rpm for 20 min at 4 °C. After discarding the supernatant, 1 mL of 75% ethanol was used to wash the RNA pellet followed by centrifugation 13,600 rpm at 4 °C for 3 min to collect and preserve the residual ethanol. The pellet was air dried in the biosafety cabinet for 5–10 min. Subsequently, the RNA pellet was dissolved by 25–100 µL of DEPC-treated water. Eventually, total RNA was quantified and qualified using a NanoDrop and Agilent 2100 bioanalyzer (Thermo Fisher Scientific, MA, USA).

### mRNA library construction

RNase H was used to remove rRNA, and DNase I was used to digest double-stranded and single-stranded DNA in total RNA. Purified RNA from previous steps was fragmented into small pieces with fragment buffer at an appropriate temperature. First-strand cDNA was generated using First Strand Master Mix by PCR, and second-strand cDNA was also generated. The reaction product was purified by magnetic beads. A-Tailing Mix and RNA Index Adapters were then added for end repair. The cDNA fragments were amplified with adapters by PCR, and the products were purified by Ampure XP Beads. The library was validated on an Agilent Technologies 2100 bioanalyzer for quality control. The double-stranded PCR product from the previous step was heat denatured and circularized by the splint oligo sequence. The single strand circle DNA was formatted as the final library. The final library was amplified with phi29 (Thermo Fisher Scientific, MA, USA) to generate DNA nanoballs (DNBs), which had more than 300 copies of one molecule. DNBs were loaded into the patterned nanoarray, and single-end 50-base reads were generated on the BGISEQ500 platform (BGI-Shenzhen, China).

### RNA-seq

The data of sequencing were screened with SOAPnuke (v1.5.2) by the following steps: (1) erasing reads having sequencing adapters; (2) removing reads that had base ratio of low-quality (base quality equal to or less than 5) more than 20%; and (3) deleting reads that had an unknown base (‘N’ base) more than 5% ratio. Then, we obtained and saved the clean reads in FASTQ format. HISAT2 (v2.0.4) were used to map the clean reads to the reference genome. The clean reads were aligned to the reference coding gene set using Bowtie2 (v2.2.5), the we calculated the expression level of the gene through RSEM (v1.2.12). According to the gene expression of different samples, the heatmap was generated by pheatmap (v1.0.8).By using DESeq2 (v1.4.5) with a Q value ≤ 0.05, differential expression analysis was performed. In order to obtain insight into the alternation in phenotype, KEGG (https://www.kegg.jp/) [15–17] and GO (http://www.geneontology.org/) enrichment analyses of annotated differentially expressed genes were carried out by phyper (https://en.wikipedia.org/wiki/Hypergeometric_distribution) based on the hypergeometric test. Through Bonferroni, the significance levels of terms and pathways were corrected by the Q value with a rigorous threshold (Q value ≤ 0.05).

### ATAC-seq

Fresh tissue sample is flash frozen by liquid nitrogen and then ground completely. The transposition reactions are initiated by adding transposase. The PCR reaction system is configured to initiate PCR amplification of the transposition products. The corresponding library quality control protocol will be selected depending upon product requirements. Single-stranded PCR products are produced via denaturation. The reaction system and program for circularization are subsequently configured and set up. Single-stranded cyclized products are produced, while uncyclized linear DNA molecules are digested. Single-stranded circle DNA molecules are replicated via rolling cycle amplification, and a DNA nanoball which contain multiple copies of DNA is generated. Sufficient quality DNA nanoballs are then loaded into patterned nanoarrays using high-intensity DNA nanochip technique and sequenced through combinatorial Probe-Anchor Synthesis.

### Adipocyte culture and differentiation

Inguinal subcutaneous adipose tissues from 3-week-old male C57BL/6J mice were removed and minced with a scalpel. After digestion with collagenase and centrifugation, the extracted primary adipocytes were cultured in growth medium [Dulbecco’s modified Eagle medium (DMEM) containing 10% fetal bovine serum (FBS)], and the medium was changed regularly for a total of 4 days. After 4 days, the cells were cultured in differentiation medium [DMEM containing 10% FBS, 1 mM dexamethasone, 0.5 mmol/L 3-isobutyl-1-methylxanthine, and 20 nM insulin]. Upon reaching 100% confluency, cells were treated with triiodothyroxine at a hypophysiological dose (0 nM), physiological dose (10 nM), and supraphysiological dose (100 nM) [18], and the medium was replaced every 2 days. Cells reached complete differentiation after a total of 6 days. The entire cycle lasted for 10 days. The methods were performed in accordance with the approved guidelines of the Animal Care and Use Committee of Nanjing Medical University. The experimental protocols were approved by the Animal Care and Use Committee of Nanjing Medical University.

### qRT‒PCR

Total RNA from the fully differentiated adipocytes was extracted using the lipid RNeasy kit and the RNeasy mini kit (Qiagen). cDNA was reverse transcribed by a transcription kit (TaKaRa) for qRT‒PCR. Gene expression was assessed by qRT‒PCR using a ViiA7 sequence detection system (Life Technologies) and TaqMan technology using the following thermocycler parameters: 1 cycle of 95 °C for 10 min; and 40 cycles of 95 °C for 10 s and 60 °C for 1 min. The sequences of the primers are shown in Supplemental Table 2. The ratio of the target gene to the copy number of the internal reference in each sample was used to represent the relative expression level of the target gene.

### Measurement of O_2_ consumption

Fresh adipose tissue was retrieved from another group of patients, the same as the sample used in the validation experiment, and was cultured in 24-well plates and differentiated as indicated (Seahorse Bioscience, North Billerica, MA). The medium was replaced with prewarmed unbuffered DMEM (DMEM basal medium supplemented with 25 mM glucose, PH7.4) and incubated at 37 °C in a non-CO_2_ incubator for 1.5 h. The oxygen consumption rate (OCR) was measured at basal glucose levels with oligomycin (ATP synthase inhibitor, 1 mM) (Sigma‒Aldrich), which disrupts the respiratory chain. The mitochondrial respiration was then blocked by 1 mM rotenone(ROT) [19] (Sigma‒Aldrich). The residual OCR was considered nonmitochondrial respiration.

### Immunofluorescence staining

Tissue sections were fixed in 4% paraformaldehyde and incubated with Mitochondria Monoclonal Antibody and ROS, MitoSOX Red (1:100, Thermo Fisher Scientific, MA, USA) at 4°C overnight followed by incubation with FITC-conjugated secondary antibody (1:100, Jackson Immuno Research Inc., West Grove, PA, USA) for 60 minutes at room temperature. 4’,6-Diamidino-2-phenylindole (DAPI; Cell Signaling Technology, Danvers, MA, USA) was used to counterstain the nuclei. Cells were visualized and imaged under 20× magnification by fluorescence microscopy (Olympus, Tokyo, Japan).

### Statistical analysis

The results are presented as the mean ± standard error of the mean (SEM). Statistically significant differences were calculated using Student’s t test. A value of P < 0.05 was considered significant. Q-value was applied in the screen of significantly enriched pathways, the Q-value is an adjusted p-value, taking in to account the false discovery rate (FDR), an FDR-adjusted p-value (aka a q-value) of 0.05 implies that we are willing to accept that 5% of the tests found to be statistically significant (e.g. by p-value) will be false positives.

## Results

### Gene expression differences in SAT from human subjects of different ages

**Fig. 1:**
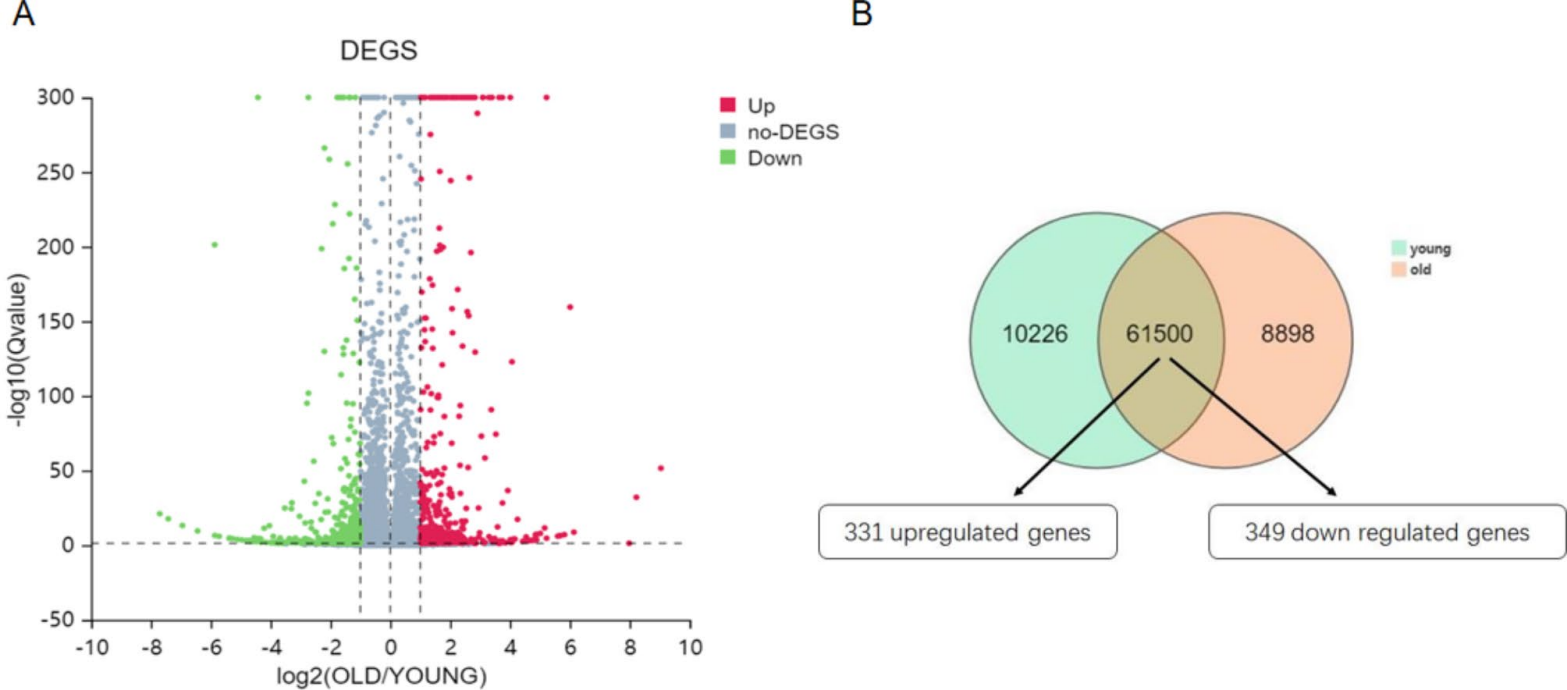
Genome characterization. (**A**) Volcano plot of differentially expressed genes. Upregulated genes are shown by red dots, and downregulated genes are shown by green dots. (**B**) Venn diagram showing 17,683 genes expressed both in the elderly and young SAT, with 331 genes upregulated and 349 genes down regulated in the common zone

**Fig. 2:**
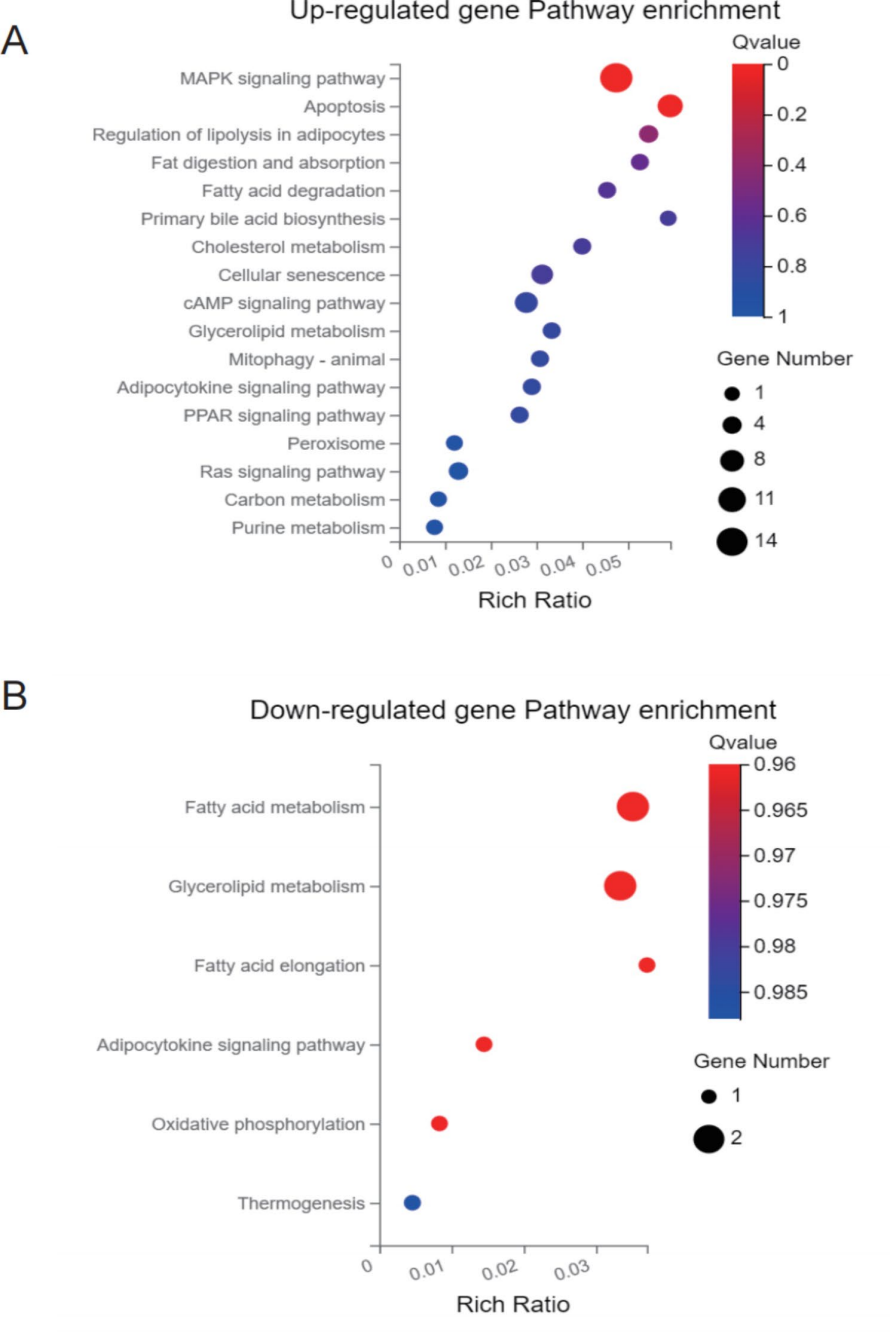
KEGG pathway enrichment analysis (**A**) Upregulated gene enrichment in signaling pathways. (**B**) Downregulated gene enrichment in signaling pathways.The enrichment factor was defined as the ratio of the number of differential genes enriched in the pathway to the number of annotated genes

**Fig. 3:**
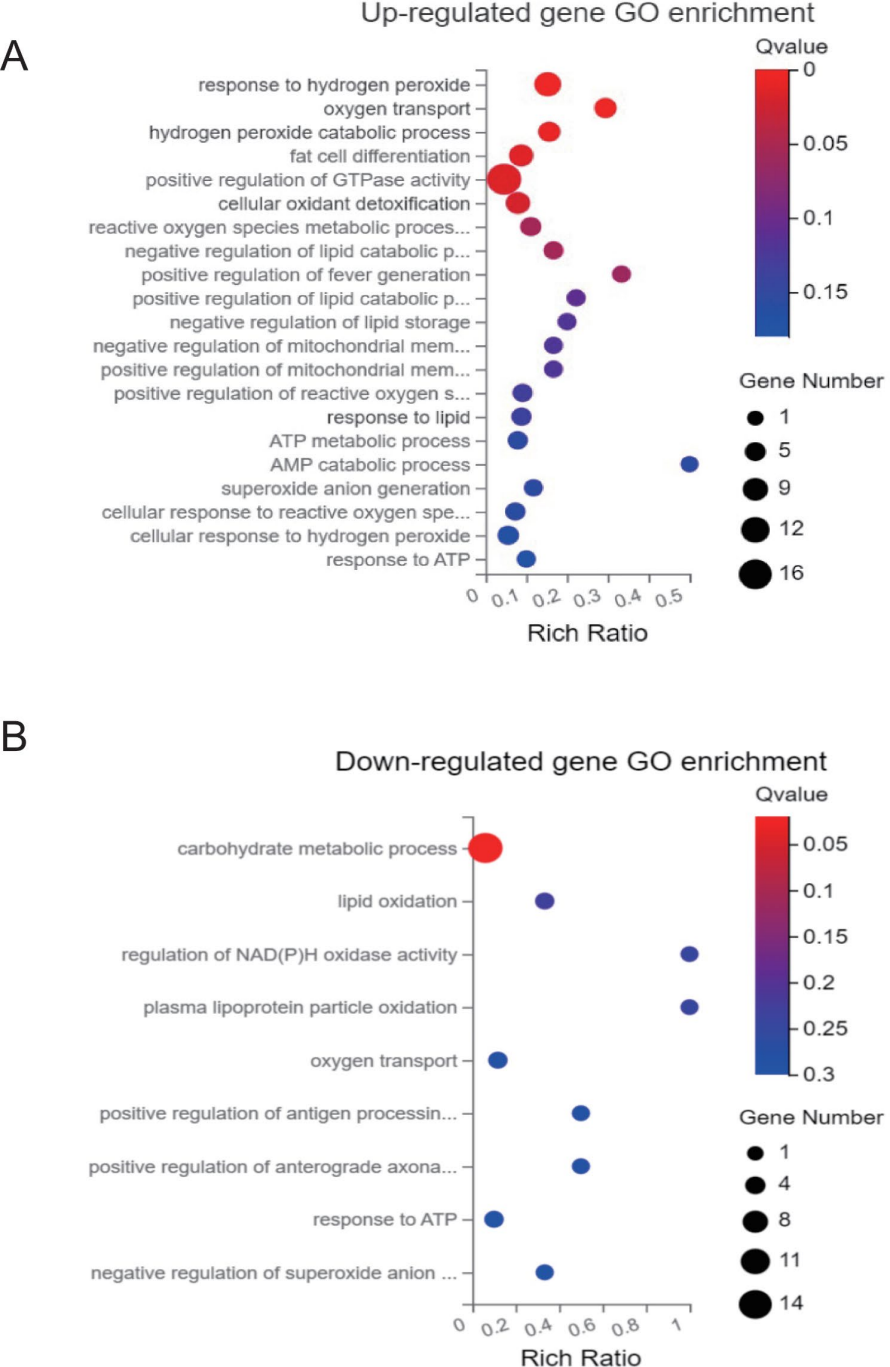
GO-P enrichment analysis (**A**) Upregulated gene enrichment in biological processes. (**B**) Downregulated gene enrichment in biological processes. The enrichment factor was defined as the ratio of the number of differential genes enriched in the pathway to the number of annotated genes

To explore the specific mechanism of how SAT affects aging, we analyzed the differential expression of RNA sequences in the SAT of 3 young men and 3 old men. A volcano plot of the expressed genes was generated (Fig. 1A), which showed differentially expressed genes in the two groups of subjects. There were 17,683 genes in both young and old SAT, among them, 331 genes showed significantly higher expression in the SAT of elderly individuals, and 349 genes showed lower expression in the SAT of the elderly (Fig. 1B). To further understand the possible functional implications of these gene expression characteristics, KEGG and GO analyses of these differentially expressed genes were performed. The upregulated differentially expressed genes of aged subcutaneous fat were enriched in the MAPK signaling pathway, lipid metabolism, and fatty acid metabolism (Fig. 2A) [15–17]. The downregulated differentially expressed genes were included in the fatty acid degradation and oxidative thermogenesis pathway (Fig. 2B) [15–17]. In addition, GO analysis showed that the upregulated genes were enriched in the processes of lipid synthesis and storage, oxygen transport, ATP generation, ROS production, and oxidative stress (Fig. 3A), while the downregulated genes participated in the processes of redox, ion transport, and lipid metabolism (Fig. 3B). Thus, these findings indicated that several functional pathways are involved in the development of aging.

### Mitochondrial function changes in aging adipose tissue

**Fig. 4:**
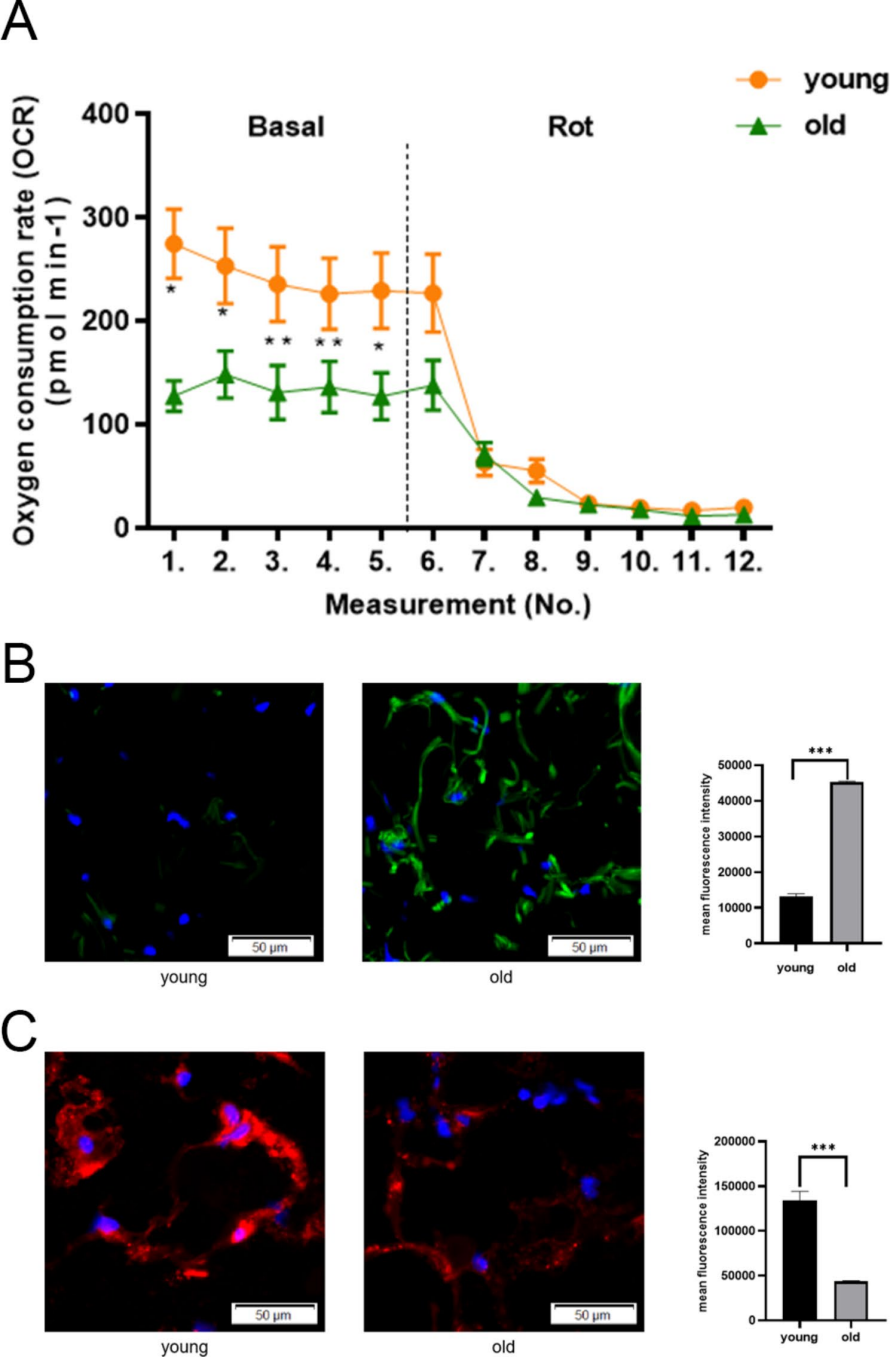
Measurement of O2 consumption and immunofluorescence staining. (**A**) OCRs were quantified in adipose tissue under basal conditions (Basal) or with rotenone (Rot) disrupting the respiratory chain. Paired t test; *p < 0.05 and **p < 0.01. (**B**) ROS in frozen sections of human subcutaneous fat were stained by immunofluorescence. Nuclei were counterstained with DAPI. Fluorescence intensity was quantified using densitometric image analysis software with cell quantity adjustment. (**C**) Mitochondria in frozen sections of human subcutaneous fat were stained by immunofluorescence. Nuclei were counterstained with DAPI. Fluorescence intensity was quantified using densitometric image analysis software with cell quantity adjustment

TO further understand the functional associated changes in SAT, considering the GO analysis results, we selected the aimed at the pathways linked with mitochondrial damage, mitochondrial dysfunction, and ROS accumulation. We first conducted an oxygen consumption experiment on SAT at different ages. Consistent with the inhibition of mitochondrial functional gene expression in aging subcutaneous adipose cells, the oxygen consumption rate (OCR) of old subcutaneous adipose cells was lower than that of young subcutaneous adipose cells under basal conditions. However, there were no significant differences in nonmitochondrial respiration [rotenone] (Fig. 4A). Immunofluorescence staining on frozen SAT sections demonstrated that old SAT contained more ROS and less mitochondria than young SAT (Fig. 4B)(Fig. 4C). Thus, these findings indicated that ROS accumulation may be related to impaired mitochondrial function in the SAT of elderly individuals.

### Characterization of the accessible chromatin profile in SAT of individuals of different ages

**Fig. 5:**
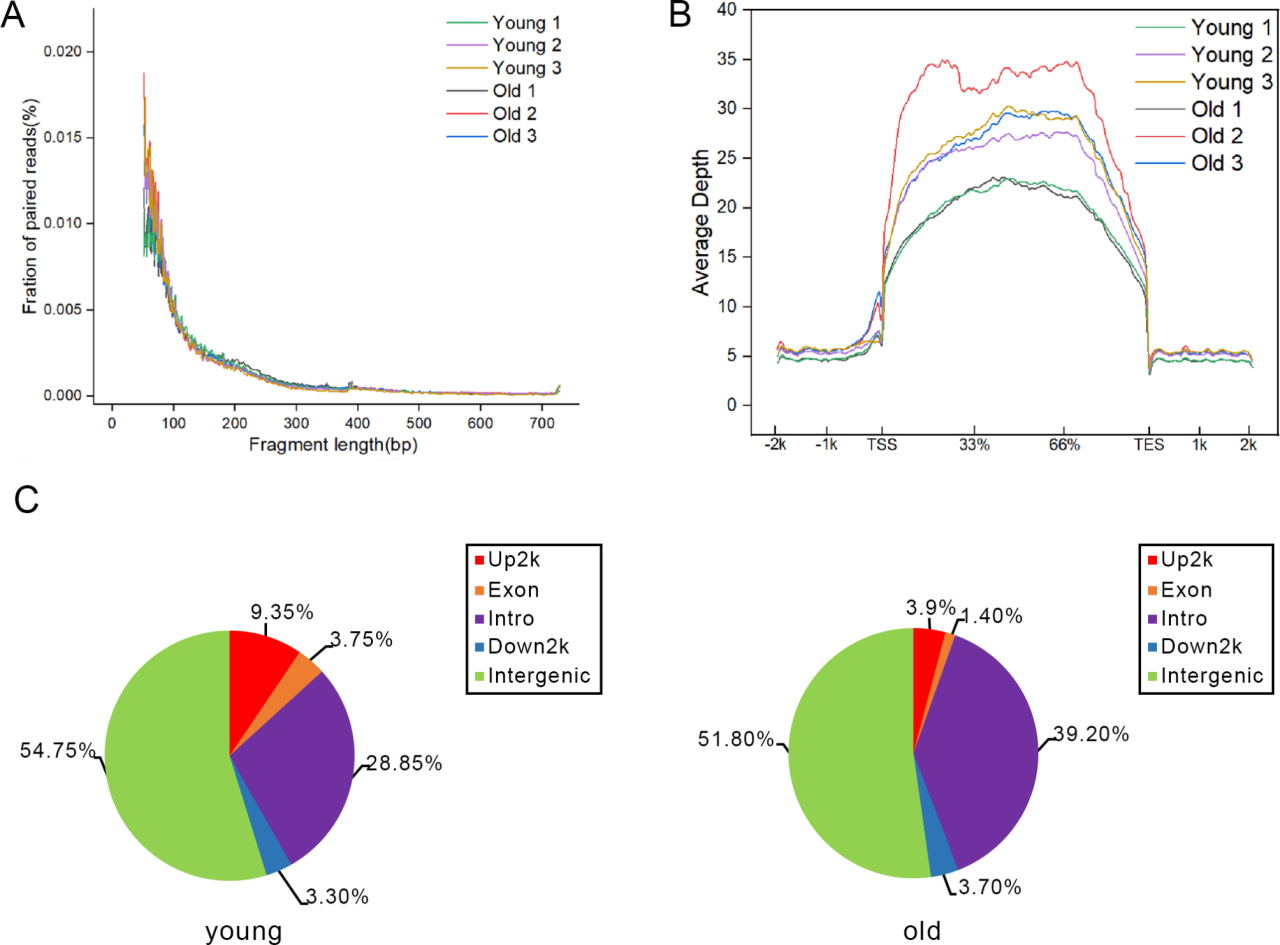
ATAC-seq chromatin accessibility analysis in young and old SAT. (**A**) Distribution of ATAC-seq fragment size in SAT. (**B**) Chromatin accessibility around the TSS in SAT. (**C**) Relative proportions of gene coding regions, intergenic regions, introns, exons, upstream regions, and downstream regions

To understand the specific mechanisms by which differentially expressed genes regulate adipose tissue, we also investigated open chromatin mass spectrometry of human SAT. To gain a genome-wide view of accessible chromatin regions from the isolated adipocytes, we utilized an assay for Transposase-Accessible Chromatin followed by sequencing (ATAC-seq). The chromatin was fragmented by Tn5 transposase into nucleosome-free, mono-nucleosome, and di-nucleosome patterns, and the similar distribution of fragment sizes suggested that chromatin was accessible to Tn5 transposase to the same degree in all samples independently among different groups (Fig. 5A). In addition, we found that the ATAC-seq signal was mainly concentrated in the intergene region (Fig. 5B). The relative enrichment ratios of coding regions, intergenic regions, introns, exons, upstream regions, and downstream regions of the SAT genome at different ages were summarized. The results showed that the proportion of the promoter region (UP2K) in the regions accessible to chromatin was altered at different ages (Fig. 5C).

### Differential gene expression in adipose tissue is associated with chromatin accessibility changes

**Fig. 6:**
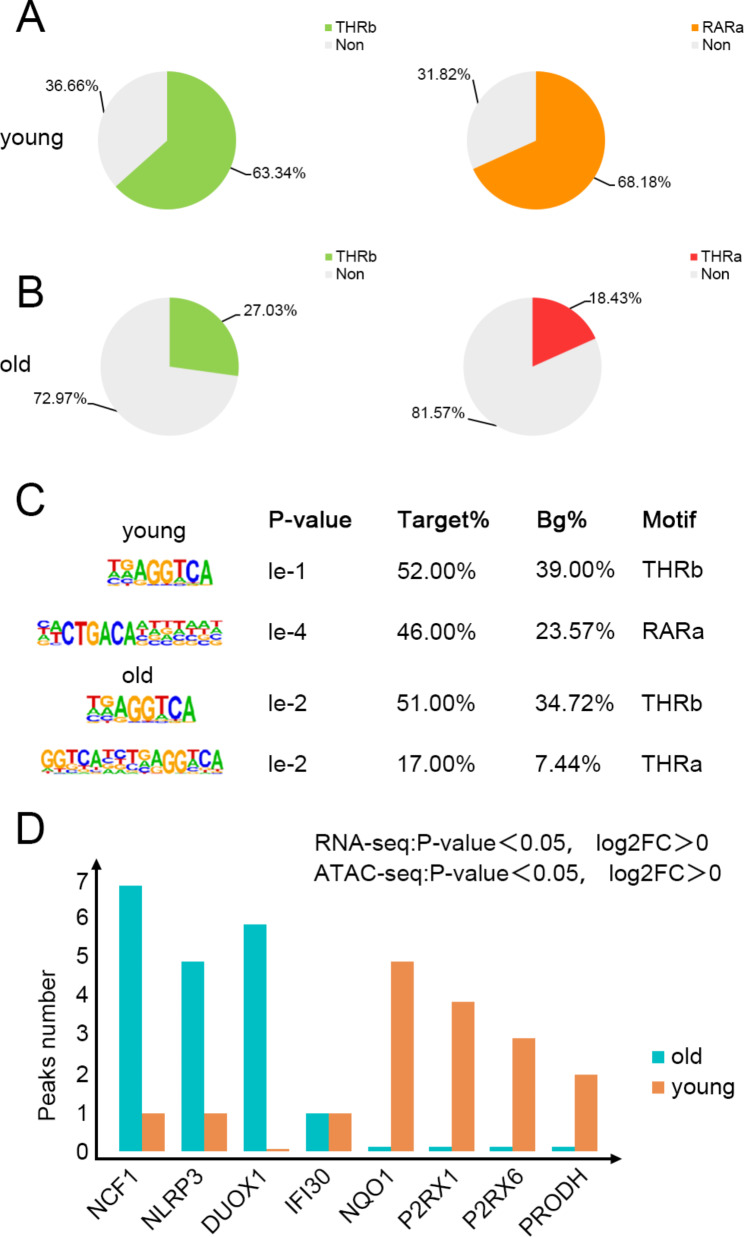
Association between the specific chromatin-accessible regions and gene expression in SAT. (**A**) Proportion of thyroid hormone receptors(THRb)and their co-transcription factor-binding sites(RARa) in the open chromatin region of related genes in the young SAT. (**B**) Proportion of thyroid hormone receptors(THRa,THRb)in the open chromatin region of related genes in the old SAT. (**C**) Enriched motif matrices are presented along with the p values. Percentages of each motif found in the target (Target %) and background (Bg%) genomic regions. (**D**) Gene-specific open chromatin regions in SAT at different ages

To examine the correlation between depot-specific open chromatin regions from the ATAC-seq analysis with depot-specific gene expression signatures, we integrated the ATAC-seq with RNA-seq datasets. Nowadays, more and more researches point out that the aging-related diseases are linked with impaired cell metabolism and mitochondrial dysfunction. As obtained from the GO analysis, the differentially expressed genes are included in the mitochondrial-related pathways which particularly aroused our interest, so we next cross-compared these genes with those genes containing thyroid hormone binding site in their open chromatin regions shown in the ATAC-seq. Eventually we concluded 7 differentially expressed genes associated with mitochondrial function, and with thyroid hormone receptors and their co-transcription factor-binding sites located in their open regions. A previous study has reported that the RARa retinoic acid receptors (RARs), a distinct class of nuclear receptors, are also efficient heterodimer partners for thyroid hormone receptors (THRs) and that RARs also serve as robust heterodimer partners and combinatorial regulators of T3-modulated gene expression [20]. In the present study, THRb and RARa accounted for a relatively high proportion in young SAT samples with approximately 63.34% and 68.18% in the open areas, respectively (Fig. 6A). At the same time, the THRb and THRa thyroid hormone receptors accounted for a relatively low proportion in old SAT samples with approximately 27.03% and 18.43% in the open areas, respectively (Fig. 6B). We also performed de novo motif analysis of specific open chromatin regions in these samples and confirmed the expression of thyroid hormone-related receptors binding sites (Fig. 6C). Cross-comparing the RNA-seq and ATAC-seq, we identified seven significant target genes as follows: the expression of Neutrophil cytosolic factor-1 (NCF1), a crucial component of nicotinamide adenine dinucleotide phosphate (NADPH) oxidase, and NOD-like receptor thermal protein domain associated protein 3 (NLRP3) which is involved in the production of ROS related inflammation, and Dual oxidase 1 (DUOX1) ,a member of the protein family of nicotinamide adenine dinucleotide phosphate NADPH oxidase, increased with age in SAT; and the expression of Interferon Gamma Inducible Protein 30(IFI30),a gene plays an important role in recycling GSH in order to neutralize ROS, and purinergic receptor P2X, ligand-gated ion channel 1 and 6 (P2RX1 and P2RX6), critical for autophagy, and Proline Dehydrogenase (PRODH), a mitochondrial flavin enzyme, decreased with age in SAT. Furthermore, the regions around these target genes gained open chromatin architecture in both young and old SAT, all of which had altered accessibility to the thyroid hormone-related receptors in their chromatin open region of different age (Fig. 6D).

### Validation of the differential expression of genes and verification of thyroid hormone regulation of target genes

**Fig. 7:**
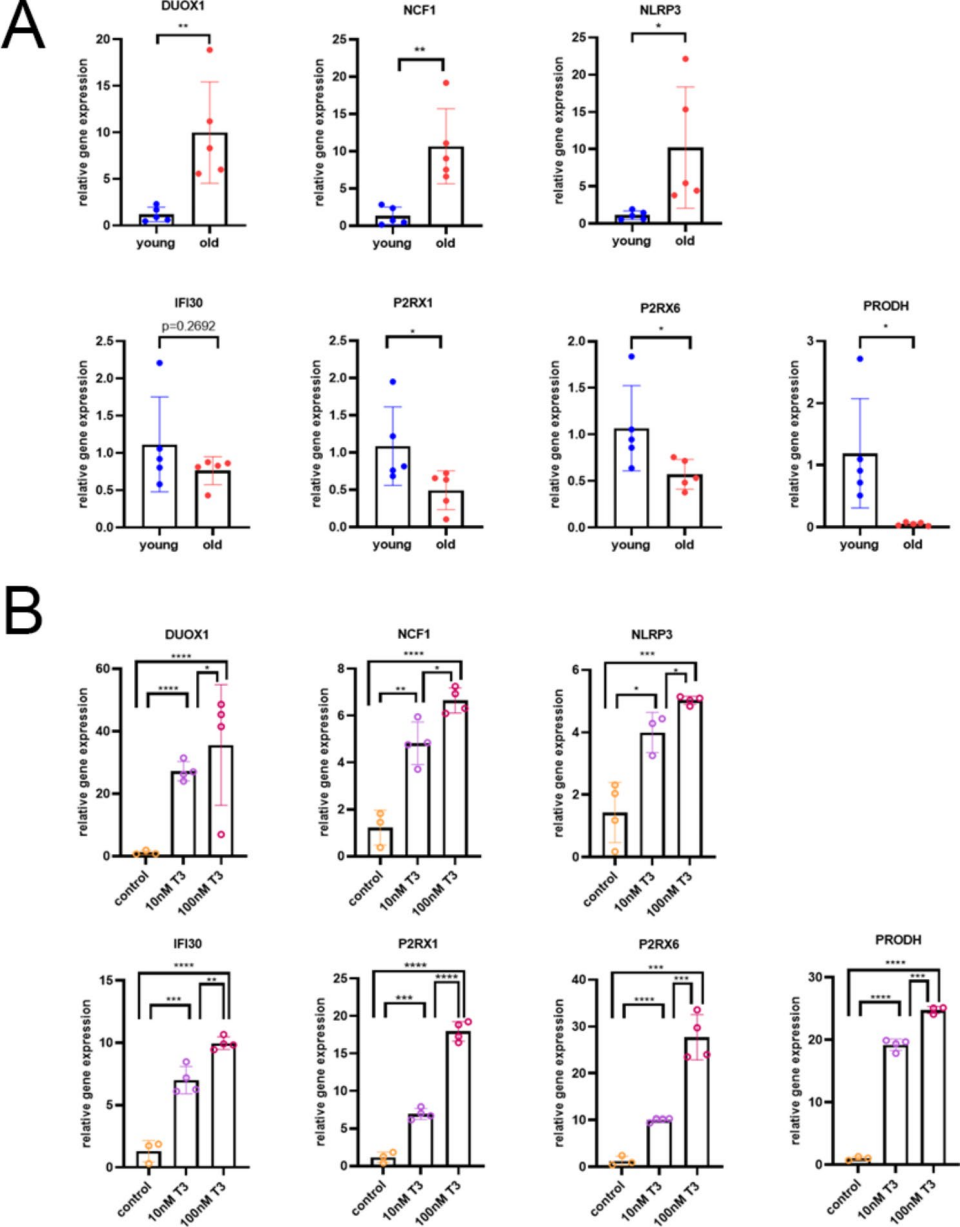
Validation of specific gene expression and its correlation with thyroid hormone regulation. (**A**) Young, subcutaneous adipose tissue of young individuals; old, subcutaneous adipose tissue of old individuals. paired t test; *p < 0.05, **p < 0.01, and ***p < 0.005. (**B**) SAT primary adipocytes of 3-week-old male C57BL/6J mice were differentiated with different concentrations of T3 (0 nM, 10 nM, and 100 nM). Relative expression of marker genes (NCF1, NLRP3, DUOX1, IFI30, P2RX1, P2RX6, and PRODH) in differentiated SVF cells from SAT (mean ± SEM; N = 4). paired t test; *p < 0.05 **p < 0.01, and ***p < 0.005

To confirm the validity of the data obtained by whole genome sequencing in these six subjects, we validated the selected genes in SAT obtained from a larger cohort of subjects (n = 5 healthy men, excluding the 6 subjects from the previous study) under the same certification of the sample used in the RNA-seq. The characteristics of the study group are shown in Supplemental Table 3. We performed qRT‒PCR on subcutaneous adipocytes to measure the mRNA levels of target genes. The expression levels of NCF1, NLRP3, and DUOX1 were increased in old subcutaneous adipocytes compared to young samples, whereas IFI30, P2RX1, P2RX6, and PRODH were highly expressed in young subcutaneous adipocytes. The results were in accordance with the RNA-seq dataset (Fig. 7A).

Next, we investigated whether these genes are regulated by thyroid hormone through the culture of primary adipocytes extracted from 3-week-old male C57BL/6J mouse SAT with different concentrations of T3 (0 nM, 10 nM, and 100 nM) throughout the entire differentiation process. Compared to the SVF cells of SAT without T3 stimulation, the gene expression in the primary cells stimulated by the physiological dose and supraphysiological dose of T3 significantly and gradually increased (Fig. 7B). Therefore, we hypothesized that adipose tissue-specific thyroid hormone signaling regulates genes involved in subcutaneous adipocyte senescence.

## Discussion

Aging is the single greatest cause of disease and death worldwide, and understanding the associated processes may vastly improve quality of life [9]. The distribution of adipose tissue significantly changes with age, and this type of adipose tissue redistribution in elderly individuals is associated with an increased risk of metabolic syndrome (diabetes, hypertension, dyslipidemia, atherosclerosis, and increased intra-abdominal fat) [4]. Using an in vivo fat transplantation strategy, some researchers have demonstrated that SAT adipose tissue has direct and beneficial effects on the control of body weight and metabolism [7]. To explore the changes that occur in SAT with age, we conducted RNA-seq on SAT at different ages. In the present study, we found that the SAT function of elderly individuals was damaged, and the pathways enriched in the GO-analysis are mainly associated with DNA damage, inflammation, fibrosis, mitochondrial damage, mitochondrial dysfunction, and ROS accumulation. Immunofluorescence staining demonstrated that mitochondria in the SAT of elderly individuals decreased and ROS accumulated. Measurement of the oxygen consumption rate (OCR) further confirmed that SAT respiration in elderly individuals was reduced. Therefore, these findings indicated that SAT undergoes mitochondrial metabolism changes during aging.

RNA-seq screened differentially expressed genes (DEGs) at the mRNA level, and these gene functions were related to mitochondrial metabolism. Although we demonstrated that the mitochondrial function of SAT changes during aging, the mechanism of aging-related changes in SAT remained unclear. To express genes, chromatin must be in an open conformation. Open chromatin allows regulatory proteins to bind to DNA and regulate DNA function. The analysis of chromatin accessibility to transposase by high-throughput sequencing (ATAC-seq) allows high-throughput sequencing of open chromatin regions with the help of transposase. ATAC-seq detects the chromatin accessibility of related genes and identifies its regulatory mechanism. At present, analysis research combining ATAC-seq and RNA-seq is not common, and such research on SAT is rare. Therefore, integration analysis allows further exploration of the key factors of biological processes and target genes of transcription factors.

Through ATAC-seq, we found that there are thyroid hormone receptor and co-transcription factor-binding sites in the open region of subcutaneous fat differential genes. Thyronine, as a predominant regulating hormone of metabolism, plays a crucial role in the turnover of mitochondria through its transcriptional effect [19]. It has been confirmed that the level of circulating T3 is correlated with energy expenditure in humans as hypothyroidism and hyperthyroidism are associated with low and high energy expenditure, respectively [21]. As age increases, the resting metabolic rate of elderly individuals decreases, and the core temperature decreases; however, the metabolism-associated thyroid hormone level does not change significantly [10]. Thus, the aging-related diseases caused by SAT may be related to the insensitivity to the thyroid hormone.

Among the DEGs associated with metabolism-related pathways, we identified seven candidate genes (NCF1, NLRP3, DUOX1, IFI30, P2RX1, P2RX6, and PRODH). NCF1, NLRP3, and DUOX1 were upregulated in SAT from elderly individuals, and as reported in the previous studies, they were enriched in ROS-related pathways, which may lead to ROS accumulation and mitochondrial dysfunction. P2RX1, P2RX6, and PRODH were downregulated in SAT from elderly individuals, and according to previous researches they exerted a protective influence on mitochondria through their roles in anti-ROS and mitochondrial repair. Thus, these findings indicated that the function of mitochondria is impaired in elderly individuals, which may be due to the changes in these genes. NCF1, NLRP3, and DUOX1 interact with NADPH oxidases, causing inflammation and accumulation of ROS in mitochondria, which is considered to be an important factor in senility and multiple diseases, leading to the infiltration of neutrophils and an increase in proteinases, thereby damaging the normal function of SAT mitochondria [22–24]. The expression of IFI30, P2RX1, P2RX6, and PRODH was decreased in elderly individuals. IFI30 plays an important role in recycling glutathione (GSH) to neutralize ROS [25]. P2RX1 and P2RX6 utilize extracellular nucleotides and adenosine as transmitter molecules, playing a critical role in regulating mitochondrial function and metabolism [26]. PRODH is a mitochondrial flavin enzyme associated with the inner mitochondrial membrane that promotes its survival [27]. To test our hypothesis, we extracted primary adipocytes from mouse SAT and stimulated them with different concentrations of T3. In accordance with our expectation, these differently expressed genes in SAT of different ages were able to be stimulated by administration of T3.

## Conclusions

In summary, based on the analysis of ATAC-seq, RNA-seq, tissue experiments, and cell experiments, we demonstrated the mitochondrial and ROS associated functional changes of SAT in the process of aging. And within the SAT of the young and the elderly, we identified seven genes whose expression are dramatically altered, further investigation were carried out on these genes, in which we found all seven of these gene’s chromatin open regions contain thyroid hormone binding site, and were able to be stimulated by TH, suggesting that thyroid signaling may be linked with the functional change of SAT in the senescence process. Due to the limitations of experimental conditions, for example, the sample content, the validation experiments performed on mice in stead of human SAT, the lack of experiments knocking out of the target genes, etc., the specific mechanisms of the seven marker genes were not determined in detail. Interestingly, accumulating evidence has associated cellular metabolism and mitochondrial dysfunction with other hallmarks of aging and age-related diseases. There is consistent evidence that age-related traits, such as increased oxidative stress and mitochondrial dysfunction, are associated with frailty [28]. Research on the involvement of thyroid hormone signaling in subcutaneous adipose tissue alteration in the aging process has a certain impact on the occurrence and development of elderly frailty.

### Electronic supplementary material

Below is the link to the electronic supplementary material.

Supplementary Material 1

## Data Availability

The datasets generated and/or analysed during the current study are available in the Mendeley data repository, [https://data.mendeley.com/datasets/52zbt6yv58], DOI: 10.17632/52zbt6yv58.1.

## Abbreviations

SAT: Subcutaneous adipose tissue
RNA: Seq:transcriptome sequencing
ATAC-seq: Assay for transposase-accessible chromatin with high throughput sequencing
AT: Adipose tissue
BMI: Body mass index
TH: Thyroid hormone
T3: Triiodothyronine
OCR: Oxygen consumption rate
RARs: RARa retinoic acid receptors
THRs: Thyroid hormone receptors
NCF1: Neutrophil cytosolic factor-1
NLRP3: NOD-like receptor thermal protein domain associated protein 3
DUOX1: Dual oxidase 1
IFI30: Interferon Gamma Inducible Protein 30
P2RX1: Purinergic receptor P2X, ligand-gated ion channel 1
P2RX6: Purinergic receptor P2X, ligand-gated ion channel 6
PRODH: Proline Dehydrogenase

## References

[1] Kalish VB (2016). Obesity in older adults. Prim Care.

[2] Batsis JA, Mackenzie TA, Barre LK, Lopez-Jimenez F, Bartels SJ (2014). Sarcopenia, sarcopenic obesity and mortality in older adults: results from the National Health and Nutrition Examination Survey III. Eur J Clin Nutr.

[3] Lv Y, Mao C, Gao X, Ji JS, Kraus VB, Yin Z, Yuan J, Chen H, Luo J, Zhou J (2022). The obesity paradox is mostly driven by decreased noncardiovascular disease mortality in the oldest old in China: a 20-year prospective cohort study. Nat Aging.

[4] Ou MY, Zhang H, Tan PC, Zhou SB, Li QF (2022). Adipose tissue aging: mechanisms and therapeutic implications. Cell Death Dis.

[5] Tran TT, Yamamoto Y, Gesta S, Kahn CR (2008). Beneficial effects of subcutaneous fat transplantation on metabolism. Cell Metab.

[6] Barrera C, Gatica A (2012). Morgan CJJobr, agents h: obese visceral adipose tissue grafted in lean mice can alter glucose homeostasis and energy efficiency. J Biol Regul Homeost Agents.

[7] Hocking SL, Stewart RL, Brandon AE, Suryana E, Stuart E, Baldwin EM, Kolumam GA, Modrusan Z, Junutula JR, Gunton JE (2015). Subcutaneous fat transplantation alleviates diet-induced glucose intolerance and inflammation in mice. Diabetologia.

[8] Porter SA, Massaro JM, Hoffmann U, Vasan RS, O’Donnel CJ, Fox CS (2009). Abdominal subcutaneous adipose tissue: a protective fat depot?. Diabetes Care.

[9] Schaum N, Lehallier B, Hahn O, Palovics R, Hosseinzadeh S, Lee SE, Sit R, Lee DP, Losada PM, Zardeneta ME (2020). Ageing hallmarks exhibit organ-specific temporal signatures. Nature.

[10] Mooradian AD (2019). Age-related resistance to thyroid hormone action. Drugs Aging.

[11] Yan Y, Niu Z, Sun C, Li P, Shen S, Liu S, Wu Y, Yun C, Jiao T, Jia S (2022). Hepatic thyroid hormone signalling modulates glucose homeostasis through the regulation of GLP-1 production via bile acid-mediated FXR antagonism. Nat Commun.

[12] de Oliveira M, Rodrigues BM, Olimpio RMC, Mathias LS, De Sibio MT, Moretto FCF, Graceli JB, Nogueira CR (2019). Adiponectin and Serine/Threonine kinase akt modulation by triiodothyronine and/or LY294002 in 3T3-L1 adipocytes. Lipids.

[13] Walczak K, Sieminska L (2021). Obesity and thyroid Axis. Int J Environ Res Public Health.

[14] Zhou Y, Yang Y, Zhou T, Li B, Wang Z (2021). Adiponectin and thyroid Cancer: insight into the association between adiponectin and obesity. Aging Dis.

[15] Kanehisa M (2000). KEGG: Kyoto Encyclopedia of genes and genomes. Nucleic Acids Res.

[16] Kanehisa M (2019). Toward understanding the origin and evolution of cellular organisms. Protein Sci.

[17] Kanehisa M, Furumichi M, Sato Y, Kawashima M, Ishiguro-Watanabe M (2023). KEGG for taxonomy-based analysis of pathways and genomes. Nucleic Acids Res.

[18] Oliveira M, Olimpio RM, Sibio MT, Moretto FC, Luvizotto Rde A, Nogueira CR (2014). Short-term effects of triiodothyronine on thyroid hormone receptor alpha by PI3K pathway in adipocytes, 3T3-L1. Arq Bras Endocrinol Metabol.

[19] Yau WW, Singh BK, Lesmana R, Zhou J, Sinha RA, Wong KA, Wu Y, Bay BH, Sugii S, Sun L (2019). Thyroid hormone (T(3)) stimulates brown adipose tissue activation via mitochondrial biogenesis and MTOR-mediated mitophagy. Autophagy.

[20] Lee S, Privalsky ML (2005). Heterodimers of retinoic acid receptors and thyroid hormone receptors display unique combinatorial regulatory properties. Mol Endocrinol.

[21] Yavuz S, Salgado Nunez Del Prado S, Celi FS (2019). Thyroid hormone action and energy expenditure. J Endocr Soc.

[22] Brunson T, Wang Q, Chambers I, Song Q (2010). A copy number variation in human NCF1 and its pseudogenes. BMC Genet.

[23] Shao BZ, Xu ZQ, Han BZ, Su DF, Liu C (2015). NLRP3 inflammasome and its inhibitors: a review. Front Pharmacol.

[24] Ashtiwi NM, Sarr D, Rada B (2021). DUOX1 in mammalian disease pathophysiology. J Mol Med (Berl).

[25] Cacialli P, Mahony CB, Petzold T, Bordignon P, Rougemont AL, Bertrand JY (2021). A connexin/ifi30 pathway bridges HSCs with their niche to dampen oxidative stress. Nat Commun.

[26] Zhuang S, Xia S, Huang P, Wu J, Qu J, Chen R, Sun N, Li D, Wu H, Zhang M (2021). Targeting P2RX1 alleviates renal ischemia/reperfusion injury by preserving mitochondrial dynamics. Pharmacol Res.

[27] Lewoniewska S, Oscilowska I, Forlino A, Palka J (2021). Understanding the role of estrogen receptor status in PRODH/POX-Dependent Apoptosis/Survival in breast Cancer cells. Biology (Basel).

[28] Barros D, Marques EA, Magalhaes J, Carvalho J (2022). Energy metabolism and frailty: the potential role of exercise-induced myokines - a narrative review. Ageing Res Rev.

