# Supplementary material for: Subcutaneous adipose tissue alteration in aging process associated with thyroid hormone signaling
Source: BMC Med Genomics. 2023 Aug 25;16:202. doi: 10.1186/s12920-023-01641-5 (PMC10463827; doi:10.1186/s12920-023-01641-5)
Supplement: Supplementary file 1 — Supplementary Material 1 [file 12920_2023_1641_MOESM1_ESM.docx]

Supplemental Table 1

Clinical parameters of men subjects (mean ± standard deviation)

|  | Clinical parameters |  | n=3 |
| --- | --- | --- | --- |
|  | Young | Old | p |
| Age (years) | 32.67±0.94 | 67.33±1.70 | <0.0001 |
| BMI (kg/m2 ) | 24.1±2.16 | 22.79±0.62 | 0.4577 |
| WHR (cm/cm) | 0.92±0.02 | 0.95±0.03 | 0.1912 |
| FT3(pmol/L) | 4.26±0.55 | 4.65±0.88 | 0.6227 |
| FT4(pmol/L) | 18.13±1.13 | 15.72±2.72 | 0.3118 |
| TSH (mUI/L) | 2.08±0.25 | 2.66±1.09 | 0.5078 |
| FPG (mmol/L) | 4.30±0.34 | 5.28±0.50 | 0.0836 |
| TG(mmol/L) | 1.52±0.98 | 1.92±0.75 | 0.6666 |
| TC(mmol/L) | 4.57±0.51 | 4.30±1.06 | 0.7641 |

- BMI, body mass index; WHR, waist-hip ratio; FT3, free triiodothyronine; FT4, free thyroxine; TSH, thyroid-stimulating hormone; FPG, fasting plasma glucose; TG, triglyceride; TC, cholesterol.
- p < 0.05 was considered statistically significant.

Supplemental Table 2

List of primer sequences used for real-time PCR

| Gene | Forward | Reverse |
| --- | --- | --- |
|  |  |  |
|  |  |  |
| Homo-DUOX1 | CCTGGCTCTAGCATGGACAC | CTGCACCTCCCACGAAATG |
| Homo-NCF1 | CAAGAGTACCGCGACAGACAT | AGGTCTTCTCGTAGTTGGCAAT |
| Homo-NLRP3 | GATCTTCGCTGCGATCAACAG | CGTGCATTATCTGAACCCCAC |
| Homo-IFI30 | CCCCTCTGCAAGCGTTAGAC | CCCGCAGGTATAGATTGCCT |
| Homo-P2RX1 | GGCTGACTACGTCTTCCCAG | GCGCAGTAGCCTTGAGTCT |
| Homo-P2RX6 | GTCCGTCCCACTGGCTAAC | CTGGCCTGTTTTTACACCGTG |
| Homo-PRODH | CCGCAGGAATGGTGTCATCA | GACTCTACCTGAGGCTTCGAT |
| Mus-DUOX1 | AAAACACCAGGAACGGATTGT | AGAAGACATTGGGCTGTAGGG |
| Mus-NCF1 | ACACCTTCATTCGCCATATTGC | CCTGCCACTTAACCAGGAACA |
| Mus-NLRP3 | ATTACCCGCCCGAGAAAGG | CATGAGTGTGGCTAGATCCAAG |
| Mus-IFI30 | CCTGGTCTCCGATCCTACCAT | TTGCAGGTGGTTGTGCCTT |
| Mus-P2RX1 | GGATGGTGCTGGTACGAAACA | CACTGACACACTGCTGATAAGG |
| Mus-P2RX6 | GTAGTCTACGTGATAGGGTGGG | CACGAGGAAGTTGGTTACCAG |
| Mus-PRODH | GCACCACGAGCAGTTGTTC | CTTTGTTGTGCCGGATCAGAG |

Supplemental Table 3

Clinical parameters of men subjects (mean ± standard deviation)

|  | Clinical parameters |  | n=5 |
| --- | --- | --- | --- |
|  | Young | Old | p |
| Age (years) | 35.8±3.20 | 75.2±5.34 | <0.0001 |
| BMI (kg/m2 ) | 25.53±2.34 | 24.06±0.83 | 0.2280 |
| WHR (cm/cm) | 0.94±0.03 | 0.98±0.03 | 0.0582 |
| FT3(pmol/L) | 4.70±0.83 | 4.39±0.78 | 0.5980 |
| FT4(pmol/L) | 18.25±2.57 | 14.53±1.60 | 0.0396 |
| TSH (mUI/L) | 2.86±1.06 | 1.81±0.54 | 0.1154 |
| FPG (mmol/L) | 4.17±0.43 | 5.00±0.60 | 0.0539 |
| TG(mmol/L) | 1.25±0.84 | 0.96±0.23 | 0.5274 |
| TC(mmol/L) | 3.99±0.88 | 3.87±1.06 | 0.8663 |

- BMI, body mass index; WHR, waist-hip ratio; FT3, free triiodothyronine; FT4, free thyroxine; TSH, thyroid-stimulating hormone; FPG, fasting plasma glucose; TG, triglyceride; TC, cholesterol.
- p < 0.05 was considered statistically significant.
